# Shoulder angle effects on neuromuscular activation and rapid force production: sEMG time–frequency and force–time analyses

**DOI:** 10.3389/fbioe.2026.1821665

**Published:** 2026-06-15

**Authors:** Salih Çabuk, Süleyman Ulupınar, İzzet İnce, Muhammet Raşit İnaç, Hasan Hüseyin Yılmaz, Osman Ateş, Cebrail Gençoğlu, Serhat Özbay, Zelal Apaydın

**Affiliations:** 1 Department of Coaching Education, Faculty of Sport Sciences, Erzurum Technical University, Erzurum, Türkiye; 2 Department of Coaching Education, Faculty of Sport Sciences, Ankara Yıldırım Beyazit University, Ankara, Türkiye; 3 Department of Coaching Education, Faculty of Sport Sciences, Atatürk University, Erzurum, Türkiye; 4 Department of Coaching Education, Faculty of Sport Sciences, İstanbul University - Cerrahpaşa, İstanbul, Türkiye; 5 Department of Physical Education and Sports, Faculty of Sport Sciences, Erzurum Technical University, Erzurum, Türkiye; 6 Department of Physiotherapy and Rehabilitation, Faculty of Health Sciences, Istanbul Yeni Yuzyil University, İstanbul, Türkiye

**Keywords:** electromyography, force-time curve, rate of force development, shoulder muscle activation, time-frequency

## Abstract

**Introduction:**

Maximal isometric shoulder force production constitutes an angle-dependent neuromuscular task; changes in shoulder elevation can meaningfully modify the relative contribution of glenohumeral and scapulothoracic musculature through concomitant alterations in moment arms, length–tension operating ranges, and the direction of the resultant force vector. Although shoulder testing across different arm elevations is widely used to infer functional capacity and rehabilitation readiness, the underlying neuromuscular strategies are still most often described using amplitude-based surface electromyography (sEMG) metrics or peak mechanical outcomes alone. However, it remains unclear how shoulder elevation angle influences time–frequency features of muscle activation and force–time characteristics.

**Methods:**

Twenty-four elite male athletes performed maximal isometric efforts. sEMG signals were analyzed wavelet-based mean frequency. Rate of force development (RFD) and mechanical impulse values were calculated from the raw force–time data across predefined intervals from contraction onset to 50, 100, 150, 200, 250 ms, and peak force.

**Results:**

Mean frequency was higher for the anterior deltoid and pectoralis major at 90° than at 135° and 180°. For the serratus anterior and infraspinatus, mean frequency was greater at 135° and 90° than at 180°. In contrast, upper and lower trapezius showed higher mean frequency at 180° than at 135° and 90°. No significant position-dependent differences were observed for the middle and posterior deltoid. Mechanically, the 180° condition showed higher force, RFD, and impulse values compared to the other angles.

**Discussion:**

Angle-dependent task sharing was observed within the shoulder complex, whereby lower elevation angles are associated with greater involvement of anterior musculature, while the overhead position is characterized by increased involvement of scapular stabilizers and higher rapid force-production outputs. These angle-specific neuromechanical patterns may contribute to more detailed shoulder profiling in elite athletes.

## 1 Introduction

In the shoulder complex, changes in angular position shape neuromuscular activation strategies by directly affecting both the length of the muscles’ moment arms and the direction of the force vectors (Ackland et al., 2008; Boettcher et al., 2010; Hecker et al., 2021; Uga et al., 2016; Ulupınar et al., 2025). The Athletic Shoulder Test (AST) stands out as a functional test protocol that evaluates shoulder stability and force production capacity through isometric force measurements at the I (180°), Y (135°), and T (90°) angular positions (Çiftçi et al., 2025; Intelangelo et al., 2024; Tooth et al., 2022; Ulupınar et al., 2025). Previous studies have primarily described angle-dependent muscle activation patterns using amplitude-based surface electromyography (sEMG) metrics normalized to maximal voluntary contraction (MVC). For example, Tooth et al. (2022) investigated angle-dependent activation patterns during the AST using EMG measurements limited to the upper trapezius (UT), lower trapezius (LT), and serratus anterior (SA). Their analysis therefore focused specifically on scapulothoracic muscle responses across the I, Y, and T positions. The findings showed that the trapezius and SA muscles exhibit activation patterns sensitive to angular position, with trapezius activation predominating in the I position, while the SA plays a more prominent role in the T position. These findings indicate that the scapular stabilizer muscles play an important role in angular position–dependent functional loading strategies (Tooth et al., 2022). However, this approach does not cover all functional components of the shoulder complex, because in addition to scapular stability, the glenohumeral joint muscles also have a decisive effect on both force production and the direction of the force vector (Tsuruike and Ellenbecker, 2022; Ulupınar et al., 2025). In the study by Ashworth et al. (2025), sEMG measurements were performed in the three AST positions and in isometric rotation tests, normalized to %MVC. Unlike previous limited approaches, this study has provided an extensive analysis covering nine shoulder and trunk muscles, including UT, anterior deltoid (AD), posterior deltoid (PD), infraspinatus (INF), latissimus dorsi, SA, pectoralis major (PM), and the left and right external obliques. The findings showed that SA activation was 159% higher in the T position than in the I position and 66% higher in the Y position than in the I position. In addition, in the T position, the median SA activation is above 200% MVC. For AD, the T position showed 74% higher activation than I. For INF, no specific percentage difference was reported among I, Y, and T (Ashworth et al., 2025b).

The rate of force development (RFD) is a key indicator of explosive strength during rapid isometric contractions and is derived from the slope of the force–time curve (Maffiuletti et al., 2016). The early phase of RFD is governed predominantly by rapid neural drive and increases in motor unit discharge rate, whereas later phases are more strongly influenced by muscle–tendon mechanical properties and maximal voluntary force capacity. Accordingly, RFD is increasingly preferred because it is often more closely associated with sport-specific and functional performance than peak force alone and is more sensitive to both acute and chronic neuromuscular changes (Aagaard et al., 2002; Buckthorpe and Roi, 2018; Folland et al., 2014; Maffiuletti et al., 2016). In parallel, wavelet-based methods have gained substantial attention for the comprehensive analysis of sEMG (Jang et al., 2025; Jankaew et al., 2025; Napoli et al., 2015; Von Tscharner, 2000). By enabling simultaneous inspection of sEMG in the time–frequency–amplitude domain, wavelet approaches capture how frequency content evolves over time and can provide complementary insight into motor unit recruitment strategies, activation intensity, and timing (Koenig et al., 2018; Peñailillo et al., 2013). Although FFT-based approaches are commonly used in isometric EMG analysis, wavelet analysis was preferred in the present study because it enables a more detailed characterization of how signal frequency content evolves over time. This feature is particularly relevant for evaluating early-phase neuromuscular responses and position-specific activation strategies across different shoulder joint angles. Wavelet-based method have been widely used to examine muscle activation patterns in dynamic tasks such as walking/running (Koenig et al., 2018), jump-landing (Jankaew et al., 2025; Kim et al., 2020), and cutting maneuvers (Beaulieu et al., 2008); have also enabled detailed evaluation of the signal’s time–frequency behavior in isometric protocols (Chowdhury et al., 2013; Coorevits et al., 2008; Murphy et al., 2023; Jankaew et al., 2025).

Maximal isometric shoulder force production constitutes an angle-dependent neuromuscular task; changes in shoulder elevation can meaningfully modify the relative contribution of glenohumeral and scapulothoracic musculature through concomitant alterations in moment arms, length–tension operating ranges, and the direction of the resultant force vector. Although shoulder testing across different arm elevations is widely used to infer functional capacity and rehabilitation readiness, the underlying neuromuscular strategies are still most often described using amplitude-based sEMG metrics or peak mechanical outcomes alone. However, it remains unclear how shoulder elevation angle influences time–frequency features of muscle activation and force–time characteristics. Thus, the purpose of this study was to utilize wavelet-based time–frequency analysis of sEMG and force–time curve metrics to evaluate and compare angle-dependent neuromechanical strategies during maximal isometric shoulder force production at three standardized arm elevation angles. It was hypothesized that shoulder elevation angle would significantly influence both muscle-specific time–frequency activation patterns and force–time characteristics, with distinct neuromechanical profiles emerging across the I, Y, and T positions.

## 2 Materials and methods

### 2.1 Subjects

The G*Power analysis used to determine the required sample size was set as follows: effect size (Cohen’s f) = 0.25, 1−β = 0.80, α = 0.05, number of groups = 1, and number of measurements = 3. The analysis indicated a minimum sample of 23 athletes; therefore, 24 elite male athletes (age: 22.88 ± 3.54 years; body height: 176.54 ± 7.757 cm; body mass: 72.58 ± 9.231 kg; body mass index: 23.24 ± 2.182 kg/m^2^; body fat: %11.61 ± 2.112 and training age: 9.08 ± 2.501 years) from different branches (boxing, kickboxing, soccer, muay thai, karate, and taekwondo) were recruited. The sample was restricted to male athletes to minimize potential biological variability related to sex-specific differences in neuromuscular activation patterns and force production characteristics. The inclusion criteria were as follows: (i) 18–30 years of age; (ii) elite status in the primary sport (currently on the roster of a national team or a top-division/professional club, or a podium finish at a national championship within the past 24 months); (iii) no chronic health conditions, and (iv) provision of written informed consent. All procedures were approved by the Scientific Research and Publication Ethics Committee of Erzurum Technical University (Meeting No: 04, Decision No: 03, Date: 06.03.2025).

### 2.2 Study design

The EMG system and the force platform (Bertec Inc., United States of America) were sampled synchronously at 1,000 Hz. To minimize noise before electrode placement on the muscles, the skin was shaved and cleansed with isopropyl alcohol to reduce impedance below 10 kΩ. Bipolar surface Ag/AgCl electrodes were positioned parallel to the muscle fiber direction at the recommended anatomical landmarks following SENIAM guidelines (Hermens et al., 1999) and previous studies (Weber et al., 2025; Yu et al., 2023); muscle-specific placements are provided in Table 1. The following muscles were included as primary outcome variables in the EMG analysis: anterior deltoid (AD), middle deltoid (MD), posterior deltoid (PD), infraspinatus (INF), pectoralis major (PM), lower trapezius (LT), upper trapezius (UT), and serratus anterior (SA).

**TABLE 1:** sEMG electrode placements.

Muscles	Electrode placements
Anterior deltoid (AD)	One finger width distal and anterior to the acromion
Middle deltoid (MD)	On the line from the acromion to the lateral epicondyle on the greatest deltoid muscle bulge in the direction of the line from the acromion to the hand
Posterior deltoid (PD)	2 cm below the posterior angle of the acromion
Infraspinatus (INF)	Center of the infraspinous fossa
Pectoralis major (PM)	In neutral arm position 3.5 cm medial to the anterior axillary line
Lower trapezius (LT)	2/3 of the way on a line from the root of the spine of the scapula to the T8 spinous process in the direction of the line between T8 and the acromion
Upper trapezius (UT)	Halfway between the acromion and the C7 spinous process in the direction of the line between the acromion and C7
Serratus anterior (SA)	Along the mid-axillary line at ribs 6–8 such that the electrodes were anterior to the latissimus dorsi muscle when the arm was flexed to 90°; oriented parallel to the direction of muscle fibers (running from the ribs to the anterior aspect of the medial scapular border)

**FIGURE 1:**
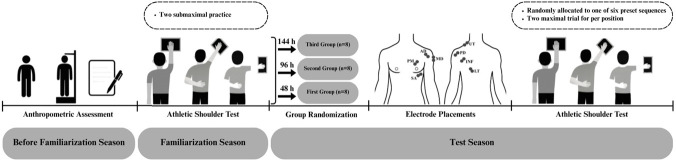
Research design of study.

Before the familiarization session, each participant’s anthropometric characteristics were recorded. After the familiarization session, participants were randomly assigned to three groups (Group 1: n = 8 Group 2: n = 8, and Group 3: n = 8). This grouping procedure was implemented to distribute the testing workload across separate sessions, thereby reducing the risk of procedural fatigue among both participants and assessors and ensuring consistent execution of the standardized AST protocol. 48-h interval was selected between familiarization and testing to reduce potential residual fatigue, learning effects, and recovery-related influences before maximal isometric efforts. Accordingly, Group 1 was tested 48 h after familiarization, Group 2 at 96 h, and Group 3 at 144 h, with 48-h intervals maintained between testing days. In each session, participants performed the AST under standardized procedures and physiotherapist supervision. To reduce order effects and enable balanced comparisons, the I, Y, and T positions were administered in a randomized, counterbalanced order rather than a fixed sequence. Participants were assigned to one of six predetermined sequences ([a] I–Y–T, [b] I–T–Y, [c] Y–I–T, [d] Y–T–I, [e] T–I–Y, and [f] T–Y–I), which were evenly distributed across groups to ensure comparable representation of each order. All measurements were conducted in the same laboratory environment, at similar times of day, and under constant environmental conditions (Figure 1).

### 2.3 Isometric shoulder force assessment

For isometric shoulder force assessment, participants were tested using AST protocol originally described by Ashworth et al. (2018) and used in previous studies (Ashworth et al., 2025a; Ashworth et al., 2025b; Ashworth et al., 2018; Çiftçi et al., 2025; Morrison et al., 2021; Tooth et al., 2022). Two maximal trials were performed per position. A 2 min rest interval was provided between repeated maximal trials within the same position, and a 30 s rest interval was provided when transitioning between the I, Y, and T positions (Ashworth et al., 2018). The highest value obtained from the two trials was used for subsequent analysis.

### 2.4 Data processing for EMG time–frequency analysis

Raw EMG signals were band-pass filtered at 20–450 Hz using a fourth-order Butterworth filter to attenuate motion artifacts and high-frequency noise. The filtered signals were full-wave rectified to enhance the representation of signal amplitude within the time–frequency domain, consistent with previous EMG wavelet-based studies (Jankaew et al., 2025; Hu et al., 2025), and subsequently processed in MATLAB (R2023b) using a custom script. Scalograms for the muscles were computed using the complex Morlet (Gabor) mother wavelet. The frequency domains of the signals were defined as in Equation 1. In this equation, H(ω) denotes the Heaviside step function, ω_0_ the dimensionless central frequency of the wavelet, and s the scale parameter.

$$\psi_{0}\left(s\omega \right)=\pi^{-\frac{1}{4}}H\left(\omega \right)e^{\frac{{-\left(s\omega -\omega_{0}\right)}^{2}}{2}}$$ (1)

The coefficients obtained from the wavelet transform were normalized using L1 normalization. Following the wavelet distribution, the result obtained with the Morlet wavelet was in the form of a complex-valued signal.

For the mean frequency calculation, the mean absolute value metric corresponding to the frequency of each wavelet data point along the scalogram in the relevant time window was used; specifically, at each time point, the mean value for the band up to half of the sampling frequency was computed using Equation 2. In this formula, $\left|x_{n}\right|$ represents the amplitude of the EMG signal in the frequency band, and N represents the length of the EMG signal window segment.

$$mean=\frac{1}{N}\sum_{n=1}^{N}\left|x_{n}\right|$$ (2)

**FIGURE 2:**
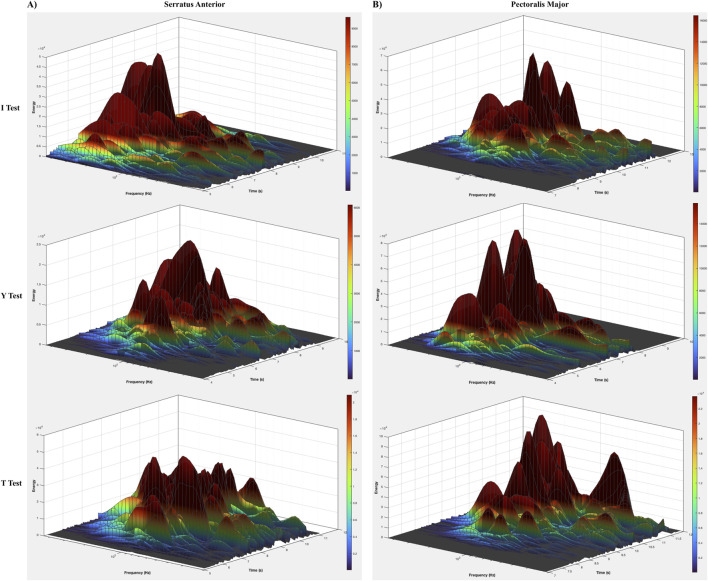
Illustrative examples of three-dimensional continuous wavelet transform plots for the serratus anterior and pectoralis major muscles across the I, Y, and T positions. **(A)** Time–frequency distribution of serratus anterior activity; **(B)** Time–frequency distribution of pectoralis major activity.

These mean absolute values corresponding to time were used to obtain the “mean frequency” curve of the signals over the time series; the summary values derived from these curves, extracted separately for each muscle, were used in the analysis as a wavelet-based characteristic feature of the respective muscle activity (Figure 2). Wavelet-based mean frequency was used to characterize muscle activity across the complete maximal isometric contraction period.

### 2.5 Data processing for force–time curve analysis

All collected force-time curves were analyzed using custom excel spreadsheet (Chavda et al., 2020). No filtering was applied to the raw force–time data (Dos' Santos et al., 2018; Guppy et al., 2022; Job III et al., 2024). Previous studies have identified force onset manually (Ashworth et al., 2025a; Guppy et al., 2022). However, this approach is sensitive to baseline noise and is known to increase variability (Guppy et al., 2024; Wang et al., 2025). As suggested in a prior study (Chavda et al., 2020), the moment at which force exceeded the quiet baseline by 5 standard deviations (SD) was accepted as the contraction onset (Yao et al., 2024). Force–time curve metrics was calculated at 50, 100, 150, 200, and 250 milliseconds from the onset of contraction.

### 2.6 Statistical analysis

All data are presented as mean (X̄) and SD. Statistical analyses were performed using R software (version 4.3.1). The normality of the data distribution was assessed using the Shapiro-Wilk test. Repeated Measures ANOVA was applied to examine the differences in force–time curve metrics and the muscles activation across different shoulder angles. When significant difference were found, *post hoc* pairwise comparisons were performed. The magnitude of the differences were calculated using Hedge’s g effect size (g) with 95% confidence intervals (95% CI). Effect sizes were classified as follows: trivial (0.00–0.19), small (0.20–0.59), moderate (0.60–1.19), large (1.20–1.99), and very large (≥2.00). A significance level of α = 0.05 was adopted for all statistical tests.

## 3 Results

**FIGURE 3:**
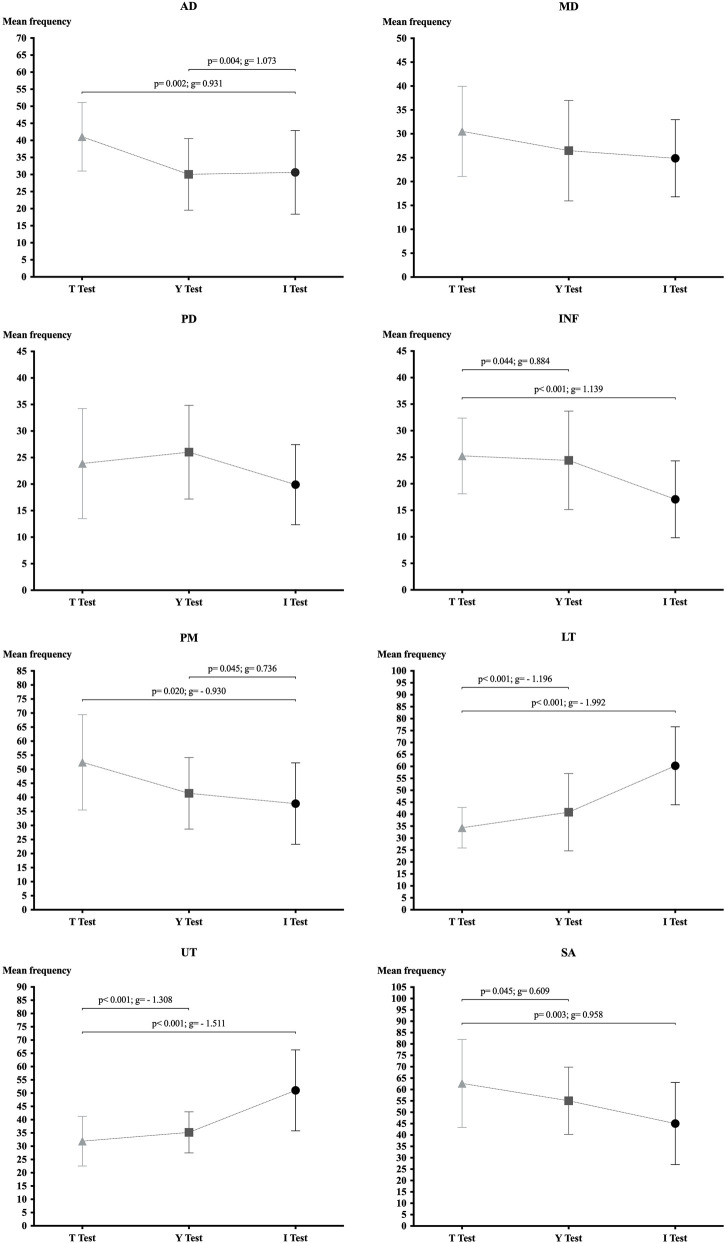
Wavelet–derived (time–frequency) muscle mean frequency across I, Y, and T positions.

The T position was associated with greater mean frequency in anterior-chain muscles, particularly the anterior deltoid and pectoralis major, whereas the Y and T positions elicited higher values for the infraspinatus and serratus anterior compared to the I position. In contrast, the I position consistently resulted in higher mean frequency for the upper and lower trapezius muscles. No significant position-dependent differences were observed for the middle and posterior deltoid (Table 2; Figure 3).

**TABLE 2:** Wavelet–based (time–frequency) muscle mean frequency across I, Y, and T positions.

Variables	I test	Y test	T-test	F_(46,2)_	*p*	η_p_^2^	Pairwise comparisons		
	X̄ (SD)	X̄ (SD)	X̄ (SD)				Positions	*p*	Hedge’s g (95% CI)
AD (Hz)	30.64 (12.256)	30.04 (10.509)	41.08 (10.067)	7.261	0.002	0.240	I vs. Y	0.999	0.053 (-0.853–0.748)
							I vs. T	0.002	0.931 (0.089–1.773)
							Y vs. T	0.004	1.073 (0.217–1.929)
MD (Hz)	24.88 (8.082)	26.47 (10.530)	30.53 (9.437)	2.167	0.126	0.086	I vs. Y	0.999	0.169 (-0.632–0.971)
							I vs. T	0.127	0.643 (-0.177–1.464)
							Y vs. T	0.554	0.406 (-0.402–1.214)
PD (Hz)	19.90 (7.545)	26.02 (8.826)	23.88 (10.364)	2.922	0.064	0.113	I vs. Y	0.062	0.745 (-0.082–1.573)
							I vs. T	0.368	0.439 (-0.371–1.249)
							Y vs. T	0.999	0.222 (-1.025–0.580)
INF (Hz)	17.06 (7.243)	24.41 (9.271)	25.25 (7.132)	6.858	0.002	0.230	I vs. Y	0.044	0.884 (0.045–1.722)
							I vs. T	<0.001	1.139 (0.277–2.002)
							Y vs. T	0.999	0.102 (-0.699–0.902)
PM (Hz)	37.82 (14.490)	41.45 (12.738)	52.48 (16.951)	6.673	0.003	0.225	I vs. Y	0.832	0.266 (-0.538–1.070)
							I vs. T	0.020	0.930 (0.087–1.772)
							Y vs. T	0.045	0.736 (-0.091–1.562)
LT (Hz)	60.28 (16.332)	40.84 (16.189)	34.36 (8.471)	25.080	<0.001	0.522	I vs. Y	<0.001	1.196 (-2.064 – - 0.327)
							I vs. T	<0.001	1.992 (-2.971 – - 1.014)
							Y vs. T	0.210	0.502 (-1.314–0.311)
UT (Hz)	51.04 (15.262)	35.21 (7.745)	31.91 (9.357)	20.858	<0.001	0.476	I vs. Y	<0.001	1.308 (-2.190 – - 0.426)
							I vs. T	<0.001	1.511 (-2.418 – - 0.604)
							Y vs. T	0.460	0.384 (-1.192–0.423)
SA (Hz)	45.03 (18.060)	55.08 (14.781)	63.10 (19.649)	7.881	0.001	0.255	I vs. Y	0.045	0.609 (-0.209–1.428)
							I vs. T	0.003	0.958 (0.113–1.802)
							Y vs. T	0.349	0.461 (-0.349–1.272)

X̄, mean; SD: standard deviation; η_p_^2^, Partial eta squared; 95% CI, 95% confidence interval; AD, anterior deltoid; MD, middle deltoid; PD, posterior deltoid; INF, infraspinatus; PM, pectoralis major; LT, lower trapezius; UT, upper trapezius, SA: serratus anterior.

**FIGURE 4:**
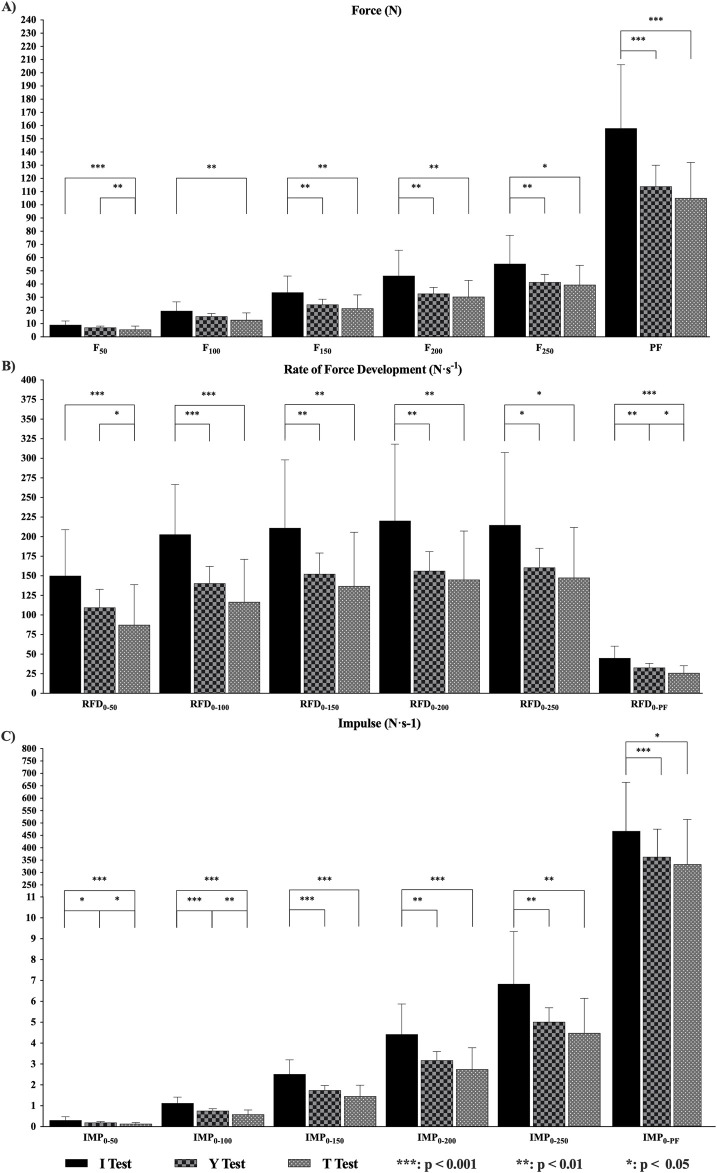
Force–time curve analysis metrics across the I, Y, and T positions: **(A)** Force at 50, 100, 150, 200, and 250 ms and peak force; **(B)** Rate of force development across the corresponding time intervals; and **(C)** Impulse across the same force–time window.

Force–time analysis revealed a clear advantage for the I position across the contraction. The I position produced higher force values than the T position at 50 and 100 m, and higher values than both the Y and T positions at 150, 200, and 250 m, as well as at peak force (Table 3; Figure 4).

**TABLE 3:** Force–time curve analysis metrics across I, Y, and T positions.

Variables	I test	Y test	T-test	F_(46,2)_	p	η_p_^2^	Pairwise comparisons		
	X̄ (SD)	X̄ (SD)	X̄ (SD)				Positions	*p*	Hedge’s g (95% CI)
F_50_ (N)	9.08 (2.990)	7.13 (2.512)	5.65 (2.518)	13.490	<0.001	0.370	I vs. Y	0.071	0.706 (-1.531–0.119)
							I vs. T	<0.001	1.241 (-2.115 – - 0.367)
							Y vs. T	0.003	0.588 (-1.406–0.229)
F_100_ (N)	19.65 (6.862)	15.57 (5.121)	12.86 (5.385)	7.813	0.001	0.254	I vs. Y	0.109	0.674 (-1.496–0.149)
							I vs. T	0.003	1.101 (-1.960 – - 0.242)
							Y vs. T	0.282	0.516 (-1.329–0.298)
F_150_ (N)	33.75 (12.277)	24.58 (9.470)	21.61 (10.263)	11.331	<0.001	0.330	I vs. Y	0.003	0.836 (-1.671 – - 0.002)
							I vs. T	0.003	1.073 (-1.929 – - 0.217)
							Y vs. T	0.646	0.301 (-1.105–0.504)
F_200_ (N)	43.37 (19.304)	32.79 (11.220)	30.42 (12.373)	11.406	<0.001	0.332	I vs. Y	0.001	0.670 (-1.492–0.152)
							I vs. T	0.007	0.799 (-1.630–0.033)
							Y vs. T	0.999	0.201 (-1.003–0.602)
F_250_ (N)	55.39 (21.521)	41.52 (13.960)	39.46 (14.752)	8.881	<0.001	0.279	I vs. Y	0.004	0.765 (-1.594–0.064)
							I vs. T	0.015	0.863 (-1.700–0.027)
							Y vs. T	0.999	0.143 (-0.945–0.658)
PF (N)	157.96 (48.241)	114.03 (37.804)	105.20 (26.885)	28.008	<0.001	0.549	I vs. Y	<0.001	1.014 (-1.864 – - 0.164)
							I vs. T	<0.001	1.351 (-2.238 – - 0.464)
							Y vs. T	0.286	0.269 (-1.073–0.535)
RFD_0-50_ (N·s^-1^)	149.95 (58.942)	109.52 (55.266)	87.36 (51.238)	11.581	<0.001	0.335	I vs. Y	0.063	0.708 (-1.532–0.117)
							I vs. T	<0.001	1.133 (-1.995 – - 0.271)
							Y vs. T	0.049	0.416 (-1.225–0.393)
RFD_0-100_ (N·s^-1^)	202.80 (63.763)	140.39 (51.277)	116.61 (54.441)	16.792	<0.001	0.422	I vs. Y	<0.001	1.079 (-1.935 – - 0.222)
							I vs. T	<0.001	1.454 (-2.353 – - 0.554)
							Y vs. T	0.400	0.450 (-1.260–0.361)
RFD_0-150_ (N·s^-1^)	211.15 (86.830)	152.43 (62.997)	136.82 (68.834)	8.912	0.002	0.279	I vs. Y	0.005	0.774 (-1.604–0.055)
							I vs. T	0.010	0.949 (-1.793 – - 0.105)
							Y vs. T	0.965	0.237 (-1.040–0.566)
RFD_0-200_ (N·s^-1^)	220.35 (97.764)	156.43 (57.996)	145.17 (61.923)	0.452	<0.001	0.291	I vs. Y	0.006	0.795 (-1.626–0.036)
							I vs. T	0.012	0.919 (-1.760 – - 0.077)
							Y vs. T	0.999	0.188 (-0.990–0.614)
RFD_0-250_ (N·s^-1^)	214.83 (92.606)	160.66 (58.123)	147.63 (63.982)	7.553	0.004	0.247	I vs. Y	0.014	0.701 (-1.525–0.124)
							I vs. T	0.022	0.844 (-1.679 – - 0.009)
							Y vs. T	0.999	0.213 (-1.016–0.589)
RFD_0-PF_ (N·s^-1^)	45.09 (15.060)	32.93 (12.466)	25.94 (9.380)	17.601	<0.001	0.434	I vs. Y	0.004	0.880 (-1.718 – - 0.042)
							I vs. T	<0.001	1.526 (-2.436 – - 0.617)
							Y vs. T	0.042	0.634 (-1.454–0.186)
IMP_0-50_ (N·s^-1^)	0.31 (0.164)	0.20 (0.112)	0.14 (0.061)	13.266	<0.001	0.366	I vs. Y	0.046	0.783 (-1.614–0.047)
							I vs. T	<0.001	1.374 (-2.264 – - 0.484)
							Y vs. T	0.025	0.665 (-1.487–0.157)
IMP_0-100_ (N·s^-1^)	1.13 (0.283)	0.76 (0.238)	0.59 (0.208)	33.914	<0.001	0.596	I vs. Y	<0.001	1.415 (-2.310 – - 0.520)
							I vs. T	<0.001	2.174 (-3.184 – - 1.165)
							Y vs. T	0.014	0.761 (-1.589–0.068)
IMP_0-150_ (N·s^-1^)	2.52 (0.685)	1.75 (0.530)	1.46 (0.527)	23.757	<0.001	0.508	I vs. Y	<0.001	1.257 (-2.133 – - 0.382)
							I vs. T	<0.001	1.734 (-2.673 – - 0.796)
							Y vs. T	0.167	0.549 (-1.364–0.266)
IMP_0-200_ (N·s^-1^)	4.42 (1.451)	3.18 (0.985)	2.75 (1.029)	15.843	<0.001	0.408	I vs. Y	0.001	1.000 (-1.849 – - 0.151)
							I vs. T	<0.001	1.328 (-2.212 – - 0.444)
							Y vs. T	0.308	0.427 (-1.236–0.382)
IMP_0-250_ (N·s^-1^)	6.84 (2.504)	5.02 (1.593)	4.49 (1.658)	11.674	<0.001	0.337	I vs. Y	0.004	0.867 (-1.704 – - 0.030)
							I vs. T	0.003	1.107 (-1.966 – - 0.247)
							Y vs. T	0.548	0.326 (-1.313–0.479)
IMP_0-PF_ (N·s^-1^)	469.06 (252.332)	358.79 (113.784)	347.40 (126.071)	5.323	0.008	0.188	I vs. Y	<0.001	0.563 (-1.379–0.252)
							I vs. T	0.020	0.610 (-1.429–0.209)
							Y vs. T	0.999	0.095 (-0.895–0.706)

X̄, mean; SD, standard deviation; η_p_^2^, Partial eta squared; 95% CI, 95% confidence interval; F_50_, Force at 50 m; F_100_, Force at 100 m; F_150_, Force at 150 m; F_200_, Force at 200 m; F_250_, Force at 250 m; PF, peak force; RFD_0-50_, Rate of force development between 0 and 50 m; RFD_0-100_, Rate of force development between 0 and 100 m; RFD_0-150_, Rate of force development between 0 and 150 m; RFD_0-200_, Rate of force development between 0 and 200 m; RFD_0-250_, Rate of force development between 0 and 250 m; RFD_0-PF_, Rate of force development between 0 and peak force; IMP_0-50_, Impulse between 0 and 50 m; IMP_0-100_, Impulse between 0 and 100 m; IMP_0-150_, Impulse between 0 and 150 m; IMP_0-200_, Impulse between 0 and 200 m; IMP_0-250_, Impulse between 0 and 250 m; IMP_0-PF_, Impulse between 0 and peak force.

A similar pattern was observed for RFD. The I and Y positions showed higher RFD values than the T position at 0–50 m, whereas the I position produced higher RFD values than both the Y and T positions at 0–100, 0–150, 0–200, 0–250 m, and 0–PF. In addition, the Y position showed higher RFD than the T position at 0–PF (Table 3; Figure 4).

Impulse outcomes showed a similar position-dependent pattern. The I position produced higher impulse than both the Y and T positions at 0–50, 0–100, 0–150, 0–200, 0–250 m, and 0–PF. Additionally, the Y position showed higher impulse than the T position at 0–50 and 0–100 m (Table 3; Figure 4).

## 4 Discussion

This study presents a position-dependent profile of muscle activation derived from time–frequency wavelet analysis and force–time curve metrics in the AST. Our findings stand out along three axes: (i) in the time–frequency domain, the T position produced higher mean frequency values for AD and PM, particularly compared to the I and Y positions, while the Y and T positions showed higher mean frequency values for INF and SA compared to the I position; (ii) for the UT and LT, higher mean frequency values were observed in the I position compared to the Y and T positions; (iii) in terms of mechanical output, the I position produced higher force, RFD, and impulse values than both the Y and T positions.

Wavelet analysis provides a critical source of information for simultaneously evaluating changes in both the temporal and frequency content of EMG signals, even in isometric test protocols such as the AST used in our study (Chowdhury et al., 2013; Coorevits et al., 2008; Murphy et al., 2023). This method allows the demonstration of the activity patterns of motor units and the neuromuscular inhibition of muscles, regulated and suppressed by the nervous system through changes in frequency, in different angular positions of the AST such as I, Y, and T (Jankaew et al., 2025; Koenig et al., 2018). The present study evaluates different isometric shoulder positions not only in terms of muscle activation or mechanical output alone, but through the combined analysis of wavelet-based sEMG mean frequency and force–time curve variables. This integrative approach allows a more detailed examination of the co-variation between neuromuscular activation patterns and mechanical outputs across the I, Y, and T positions. While most of the AST literature has reported muscle activation using amplitude-based metrics (Ashworth et al., 2025b; Tooth et al., 2022) to our knowledge, no study has compared the I, Y, and T positions by jointly considering time–frequency methods and force–time derivatives. As a natural consequence of this original design, a direct one-to-one comparison of our findings with previous studies is not possible.

The T position produced significantly higher mean frequency values for AD and PM compared with the I and Y positions. This finding is partly consistent with previous AST research. For example, Ashworth et al. (2025) reported that the T position imposed a high demand on anterior-chain muscles in baseball players, with AD activation being 74% higher in the T position than in the I position (Ashworth et al., 2025b). However, comparisons with studies involving overhead or racquet-sport athletes should be interpreted with caution, because the present sample consisted primarily of combat-sport athletes, who may exhibit different upper-extremity loading patterns, movement demands, and chronic neuromuscular adaptations. Therefore, although the similarity between studies may reflect shared biomechanical constraints of the AST positions, the direct transferability of findings across sport-specific populations remains limited. From a biomechanical perspective, the high mean frequency observed in the T position may be related to its specific mechanical configuration. In the original AST protocol, the T position is performed at 90° shoulder abduction, with forearm pronation and full elbow extension. In this prone position, applying vertical force to the force platform requires strong horizontal adduction at the glenohumeral joint (Ashworth et al., 2025a; Ashworth et al., 2025b; Ashworth et al., 2018; Dyrna et al., 2018). Thus, the T position may increase the neuromuscular demand placed on the anterior chain. Nevertheless, these frequency-based findings should be interpreted within the mechanical context of the AST positions. Changing the shoulder position from 180° to 90° may substantially alter muscle length, moment arm, and force-vector orientation, and these angle-dependent mechanical changes can influence muscle fiber conduction velocity and, consequently, sEMG mean frequency independently of neural drive. Therefore, the higher mean frequency observed in the T position likely reflects the combined influence of neural activation demands and position-dependent peripheral muscle properties.

Tooth et al. (2022) examined three scapulothoracic muscles and reported the highest %MVC for the SA, particularly in the T position, suggesting greater SA loading at 90° abduction with a more horizontal adduction–oriented force vector (Tooth et al., 2022). That is, when the arm was positioned closer to the trunk at 90° abduction and the force vector was directed more toward horizontal adduction, the loading level of the SA muscle increased significantly. Ashworth et al. (2025) used the AST protocol to evaluate shoulder function and comparatively examined the activation levels of eight muscles in the AST positions. The findings showed that, especially in the T position, SA activation was 159% higher than in the I position and 66% higher than in the Y position (Ashworth et al., 2025b). Similarly, in tennis players, Escamilla and Andrews (2009) similarly observed higher SA activation during the forehand than during the serve, indicating greater scapulothoracic demand in this plane (Escamilla and Andrews, 2009). The literature has shown that, at angles similar to the T position, SA reaches high activation levels in both isometric (Ashworth et al., 2025b; Tooth et al., 2022) and dynamic tasks (Escamilla and Andrews, 2009); this reveals a pattern consistent with our wavelet-based mean frequency analyses. The main possible reason underlying this may stem from the unique biomechanical demands of this position.

In particular, the SA stands out as a critical muscle for stabilization of the upper extremity in the horizontal plane and for the transfer of force production to the trunk in open kinetic chain tasks (Kim et al., 2019; Mendez-Rebolledo et al., 2022; Park and Yoo, 2011; Uga et al., 2016). Considering that, in the T position, the arm is positioned closer to the trunk with 90° abduction and parallel to the axis of gravity, that scapular protraction is basically dominant, and that it is an isometric variation of the pushing action requiring the application of a vertical force against the ground (Ashworth et al., 2025a; Ashworth et al., 2025b; Ashworth et al., 2018; Dyrna et al., 2018; Tooth et al., 2022; Ulupınar et al., 2025), this configuration may increase the contribution of SA to scapular stability. In addition, this increase in activation may be clinically meaningful for evaluating compensatory movement strategies observed in pathological conditions. Because in scapular dyskinesia or shoulder pathologies, weak activation of the SA often leads to suboptimal scapular control, which can trigger shoulder dysfunctions (Diederichsen et al., 2009; Ekstrom et al., 2003; Kim et al., 2019; Ng and Wu, 2021; Weber et al., 2025). Therefore, low SA activation observed in the T position may warrant further investigation as a potential marker in rehabilitation contexts. (Weber et al., 2025).

The scapular stabilizer muscles UT and LT (Ashworth et al., 2025b; Kibler and Sciascia, 2019; Uga et al., 2016) exhibited significantly higher mean frequency values in the I position compared to the Y and T. This finding may suggest greater neuromuscular demand on the scapulothoracic complex in the 180° overhead position. Biomechanically, this is quite consistent: the full overhead position requires the scapula to achieve maximal upward rotation, and UT, LT, and SA form the primary force couple that produces this rotation (Braman et al., 2009; Kim et al., 2022; Neumann and Camargo, 2019). In the prone position, locking the scapula in this end–range position and preventing it from being pulled downward while applying maximal isometric force against the force plate on the ground (Ashworth et al., 2025a; Ashworth et al., 2025b; Ashworth et al., 2018) can create a very high neuromuscular demand on UT and LT. The increase in mean frequency observed in the wavelet technique analysis is a reflection of this demand in time–sensitive neural activation strategies. This finding meaningfully complements previous EMG-based studies in the AST literature. Similarly, in the study by Tooth et al. (2022), trapezius muscle activation became dominant in the I position, and this position significantly increased the demand for scapular stabilization In addition, the findings reported by Ashworth et al. (2025) support that the high frequency values observed in the I position in the UT and LT muscles may be a position-sensitive indicator of the neuromuscular recruitment capacity of the scapulothoracic muscles (Ashworth et al., 2025b). The I position appears to be a key biomechanical configuration for assessing both overhead upper-extremity force capacity and the neural demands of scapular stabilization. As the test shifts toward the Y and T positions and the requirement for scapular upward rotation decreases, stabilization demands likely redistribute across different muscle groups, which may account for the observed differences in frequency patterns. Accordingly, the I position may be particularly informative for evaluating scapular stability and scapulothoracic muscle function in performance testing and rehabilitation.

The I and Y positions were found to produce higher values than the T in both F (F_50_) and RFD (RFD_0–50_). In the subsequent time windows (F_100_, F_150_, F_200_, F_250_, and PF), the I position produced higher force than Y and T positions. Similarly, in the corresponding windows of RFD, the I position produced a higher RFD than Y and T. In terms of mechanical impulse, the hierarchy across all windows is I > Y > T; moreover, in the early phase (IMP_0–50_ and IMP_0–100_), the Y position produced significantly higher impulse than T position. The literature on comparing RFD, impulse, and force outputs across positions in the AST (Ashworth et al., 2025a) is quite limited, which highlights another original aspect of our study. To our knowledge, the only study that directly performed this comparison is Ashworth et al., whose sample consisted of baseball athletes; by defining early RFD as 0–100 m, they showed that both PF and early RFD were significantly higher in the I position compared to the Y and T positions (PF = 25% higher, early RFD = 40% higher) (Ashworth et al., 2025a). The directional pattern of our findings for the early phase of contraction shows a convergence consistent with that study in terms of the superiority detected in favor of the I position. It is thought that these marked RFD and force observed between positions arise from a combination of muscular activation strategies and position–specific biomechanical advantages. RFD, especially in the early 50–75 m phase of contraction, depends largely on the “capacity to rapidly generate maximal voluntary activation,” and specifically on the “high rate of motor unit discharge” (Maffiuletti et al., 2016). The biomechanical basis of this neural advantage may lie in the length–tension relationship of the force–producing muscles. During the AST, force is produced by the downward pushing of the arms (Ashworth et al., 2025a; Ashworth et al., 2018; Çiftçi et al., 2025). One possible explanation is that the I position may provide a more favorable mechanical configuration for force production, potentially supporting more effective force generation during the early phase of contraction. Accordingly, RFD is largely determined by the interplay between rapid muscle activation and intrinsic contractile properties (Maffiuletti et al., 2016).

From an applied perspective, the position-dependent differences observed in this study indicate that the shoulder complex does not produce a uniform neuromechanical response across different angular configurations. At lower elevation angles, the more pronounced responses of anterior-chain muscles such as the anterior deltoid and pectoralis major suggest that these positions may more strongly reflect demands associated with horizontal adduction and anterior shoulder stabilization. In contrast, the higher mean frequency values observed in the upper and lower trapezius muscles, together with the increases in force, RFD, and impulse outputs at the 180° overhead position, indicate that this configuration imposes greater demands on scapulothoracic stabilization, upward rotation control, and rapid force production. Therefore, in shoulder assessments, different elevation angles should not be considered merely as geometric variations in position, but rather as angle-specific loading conditions that reveal distinct functional components of the shoulder complex. However, these interpretations are limited to isometric conditions and require further validation within dynamic and sport-specific movement contexts.

The findings of this study should be evaluated within certain limitations that must be considered. First, the study sample consisted of 24 elite male athletes from different sports. Therefore, the observed neuromuscular activation patterns and force–time characteristics should not be directly generalized to female athletes, the general population, or non-athletic clinical populations. Moreover, the heterogeneity of the sample in terms of sport background should be considered, as combat sports and soccer impose different upper-extremity demands and may lead to distinct chronic neuromuscular adaptations. Thus, sport-specific loading characteristics may have influenced the neuromuscular strategies observed in the present study. Second, all measurements were conducted under isometric conditions. Although isometric testing provides controlled and reliable assessment of shoulder force production, it may not fully reflect the neuromuscular demands of dynamic sport-specific movements involving concentric, eccentric, and stretch–shortening cycle actions. Therefore, the generalizability of these findings to real-world athletic performance should be interpreted with caution. Third, some key muscles known for the rotator cuff’s role in force production or central importance in the transfer of force to/from the trunk (Escamilla and Andrews, 2009; Kibler et al., 2006) were outside the scope of the assessment. This creates a limitation in presenting a holistic picture of shoulder stabilization. Fourth, as with all surface EMG studies, potential signal cross-talk and the complexity of interpreting frequency-based metrics should be considered when interpreting the neuromuscular findings. Fifth, the applied 20–450 Hz band-pass filtering range is commonly accepted for sEMG analysis and is appropriate for preserving the main physiological frequency components of the signal. However, considering the 1,000 Hz sampling frequency, the 450 Hz upper cutoff remains relatively close to the Nyquist limit; therefore, future studies may benefit from higher sampling rates to provide a wider methodological margin in time–frequency analyses. Future research should relate mean frequency and RFD metrics to sport-specific performance outcomes (e.g., striking) and examine whether these angle-dependent neuromechanical patterns are consistent during dynamic athletic tasks.

## 5 Conclusion

This study aimed to examine position-dependent neuromuscular demands differences by concurrently analyzing (i) muscle activation and (ii) force–time curve–derived variables across the different arm elevation angles. Mean frequency indicated clear angle-specific activation strategies: anterior deltoid and pectoralis major showed the highest mean frequency at 90°, whereas the serratus anterior and infraspinatus demonstrated greater mean frequency at 135° and 90° than at 180°. In contrast, upper and lower trapezius exhibited the highest mean frequency at 180°, suggesting increased scapular stabilizer demand in the overhead position. Mechanically, the 180° condition consistently demonstrated higher force, RFD, and impulse values compared with the 135° and 90° conditions across the analyzed contraction intervals. Collectively, these findings indicate angle-dependent task sharing within the shoulder complex, whereby lower elevation angles are associated with greater involvement of anterior musculature, while the overhead position is characterized by increased involvement of scapular stabilizers and higher rapid force-production outputs. These angle-specific neuromechanical patterns may contribute to more detailed shoulder profiling in elite athletes. However, their application to rehabilitation progression, performance monitoring, and return-to-sport decision-making should be interpreted cautiously and requires further validation, particularly given the isometric nature of the protocol and the specific characteristics of the study sample.

## Data Availability

The raw data supporting the conclusions of this article will be made available by the authors, without undue reservation.
